# Editorial

**Published:** 1890-06

**Authors:** 


### Editorial.

### A PLEA FOR THE CHILDREN OF THE PUBLIC SCHOOLS SOUTH.

It has been charged that every man who has taught school three months suffers with a chronic and incurable case of *big head,* on all school subjects. The humble writer was a pedagogue forty years ago, and *he* may possibly be still suffering from the early effects of this *disease.* Nevertheless we claim to belong to the progressive class of educational promoters, and we feel that it is our duty to speak out when there is anything in conflict with the educational interests. The art of teaching has been greatly perfected; the text books for the most part, meet the requirements of the age—and the school system has been much improved in the last decade. All of this is highly satisfactory—we feel a just pride in our public schools, colleges and universities. Our complaint is that the children of the public schools South do not have sufficient time for rest and recreation during the hot season. They should not be in school during June, July and August. The relaxing effects of summer heat, coupled with the worry of preparing recitations and the performance of the usual duties of the school room, lay the foundation for enfeebled powers of digestion, and therefore weakened constitutions. Children thus circumstanced become an easy prey to prevailing diseases, with diminished chances for recovery. The nervous system in childhood is easily upset, especially in case of females, arriving at the age of puberty. Convulsions are common. Relax the system of a child, and tax the brain with study, and other school duties, and if it escapes harm, it is sheer accident. One of my own children came near dying, a few years ago, of too much heat and too much school. We are alarmed when we see the dangers which lie in wait for the children of the public schools, especially of our large cities, during the hot months—In one case there may be only lassitude and headache; in another mental inertia; in another weak or imperfect vision. Irregular muscular action may produce lateral curvature of the spine, or develop St. Vitus dance. Can we afford to take the risk of so great a sacrifice? We think not; we owe too much to ourselves, our children, and coming generations to do so—our boards of education should not allow the schools to continue longer than nine months in the year. The usual holidays should be given. The salaries of teachers should suffer no diminution, for we will gain time, by giving time to those noble men and women who are daily wearing life away, in the pursuit of their great vocation.—G.

### DR. J. WELLINGTON BYERS.

We are gratified to announce that we have obtained the services of Dr. J. Wellington Byers, of Charlotte, N. C., for our editorial staff. Dr. Byers is one of the most cultured, experienced and versatile of the South's medical *literati*, and we bespeak for him a hearty co-operation and attention from our readers.

### LEGAL RESTRICTIONS UPON CONSUMPTION.

With the overwhelming evidences accumulating upon every hand in regard to the contagious character of phthisis we have reached a stage where it is imperatively necessary that some action should be taken towards the enactment of legal restrictions preventing its spread through such well recognized foci of infection as sleeping in passenger cars, hotels and similar places. No one pretending to the most superficial knowledge of the etiological factors of this disease can fail to perceive the important signification of such a law.

The hundreds, nay thousands of phthisical invalids who annually journey to Florida and other southern States, must, owing to the dust and irritation of travel, necessarily expectorate volumes of dangerous sputa, containing myriads of the deadly bacilli, upon the carpet, floors, seats, beds and other points of contact. This of course dries when it lodges and becomes a source of possible contagion to some susceptible subject. It is clearly within the purpose and duty of the State to prevent these nuisances and to compel their abatement as injurious to life and happiness. A law enforcing thorough and periodical cleansing and disinfection of cars, hotels etc., would accomplish this. In all well recognized and admitted contagious diseases the State interferes to protect the community and there should be an effort made in this, the most deadly of all diseases. B.

### BUFFALO LITHIA SPRINGS OF VIRGINIA.

### USE OF THIS WATER AFTER LAPAROTOMY AND SURGICAL OPERATIONS GENERALLY.

HUNTER McGUIRE, M. D., LL. D.
Late Professor of Surgery, Medical College of Virginia.

From the Virginia Medical Monthly for April, 1890.

RICHMOND, VA., March 22, 1890.

DEAR DR. EDWARDS:—I have just received your letter of this date. I use Buffalo Lithia Water very freely in my Hospital. After every case of Laparotomy I give this water for its diuretic properties, and because the stomach bears it so well—often retaining it when everything else is rejected. Indeed, I use it freely after nearly all my surgical operations. It is especially valuable in Suprapubic Cystotomy. Many years' experience in its use only confirms the good opinion I have so often expressed in regard to it.

Yours, very truly,
HUNTER McGUIRE,

### THE WILLIAM F. JENKS MEMORIAL PRIZE.

*The Second Triennial Prize, of Four Hundred and Fifty Dollars, under the Deed of Trust of Mrs. William F. Jenks, will be awarded to the author of the best essay on*

"THE SYMPTOMATOLOGY AND TREATMENT OF THE NERVOUS DISORDERS FOLLOWING THE ACUTE INFECTIOUS DISEASES OF INFANCY AND CHILDHOOD."

The conditions annexed by the founder of this prize are, that the "prize or award must always be for some subject connected with Obstetrics, or the Diseases of Women, or the Diseases of Children;" and that "the Trustees, under this deed for the time being, can, in their discretion, publish the successful essay, or any paper written upon any subject for which they may offer a reward, provided the income in their hands may, in their judgment, be sufficient for that purpose, and the essay or paper be considered by them worthy of publication. If published, the distribution of said essay shall be entirely under the control of said Trustees. In case they do not publish the said essay or paper, it shall be the property of the College of Physicians of Philadelphia."

The prize is open for competition to the whole world, but the essay must be the production of a single person.

The essay, which must be written in the English language, or if in a foreign language, accompanied by an English translation, should be sent to the College of Physicians of Philadelphia, Pennsylvania, U. S. A., before January 1, 1892, addressed to Louis Starr, M. D., Chairman of the William F. Jenks Prize Committee.

Each essay must be distinguished by a motto, and accompanied by a sealed envelope bearing the same motto and containing the name and address of the writer. No envelope will be opened except that which accompanies the successful essay.

The Committee will return the unsuccessful essays if reclaimed by their respective writers, or their agents, within one year.

The Committee reserves the right not to make an award if no essay submitted is considered worthy of the prize.

---

CHRONIC PROSTATITIS.—Professor Kobner states in the *Therapeutische Monatschrift* that salves or suppositories do not act as well as injections in cases of chronic prostatitis. He gives small rectal injections of the following:

| | | |
|---|---|---|
| ℞ | Kali iodidi, - - - | gr. xlv. |
| | Kali bromidii, - - - | gr. xxxviii. |
| | Ext. belladonnæ, - - | gr. v. |
| | Aquæ, - - - - - | ℥ vss. |

M. S. About a half ounce of this is added to two ounces of warm water and injected once or twice daily. Later on from three to five drops of tincture of iodine may be added to each injection.—*St. Louis Med. and Surg. Journal.*

